# General Guidelines for Sample Preparation Strategies in HR-µMAS NMR-based Metabolomics of Microscopic Specimens

**DOI:** 10.3390/metabo10020054

**Published:** 2020-01-30

**Authors:** Covadonga Lucas-Torres, Thierry Bernard, Gaspard Huber, Patrick Berthault, Yusuke Nishiyama, Pancham S. Kandiyal, Bénédicte Elena-Herrmann, Laurent Molin, Florence Solari, Anne-Karine Bouzier-Sore, Alan Wong

**Affiliations:** 1 NIMBE, CEA, CNRS, Université Paris-Saclay, CEA Saclay, 91191 Gif-sur-Yvette, France; thierry.bernard@cea.fr (T.B.); gaspard.huber@cea.fr (G.H.); patrick.berthault@cea.fr (P.B.); 2 JEOL RESONANCE Inc., Musashino, Akishima, Tokyo 196-8558, Japan; yunishiy@jeol.co.jp; 3 RIKEN-JEOL Collaboration Center, Yokohama, Kanagawa 230-0045, Japan; 4 Univ Grenoble Alpes, CNRS, INSERM, IAB, Allée des Alpes, 38000 Grenoble, France; pancham-singh.kandiyal@univ-grenoble-alpes.fr (P.S.K.);; 5 Univ Lyon, Université Claude Bernard Lyon 1, CNRS UMR 5310, INSERM U 1217, Institut NeuroMyoGène, 69008 Lyon, France; laurent.molin@univ-lyon1.fr (L.M.); florence.solari@univ-lyon1.fr (F.S.); 6 Centre de Résonance Magnétique des Systèmes Biologiques, CNRS-Université de Bordeaux, UMR5536 Bordeaux, France; anne-karine.bouzier-sore@rmsb.u-bordeaux.fr

**Keywords:** high-resolution magic angle spinning, NMR, microscopic samples, metabolomics, sample preparation

## Abstract

The study of the metabolome within tissues, organisms, cells or biofluids can be carried out by several bioanalytical techniques. Among them, nuclear magnetic resonance (NMR) is one of the principal spectroscopic methods. This is due to a sample rotation technique, high-resolution magic angle spinning (HR-MAS), which targets the analysis of heterogeneous specimens with a bulk sample mass from 5 to 10 mg. Recently, a new approach, high-resolution micro-magic angle spinning (HR-μMAS), has been introduced. It opens, for the first time, the possibility of investigating microscopic specimens (<500 μg) with NMR spectroscopy, strengthening the concept of homogeneous sampling in a heterogeneous specimen. As in all bioanalytical approaches, a clean and reliable sample preparation strategy is a significant component in designing metabolomics (or -omics, in general) studies. The sample preparation for HR-μMAS is consequentially complicated by the μg-scale specimen and has yet to be addressed. This report details the strategies for three specimen types: biofluids, fluid matrices and tissues. It also provides the basis for designing future μMAS NMR studies of microscopic specimens.

## 1. Introduction

Sample preparation is an essential component in metabolomics [1,2]. It requires dedication in designing protocols for precise and reliable acquisition of data with the appropriate analytical platform (i.e., gas (GC) [3] or liquid (LC) chromatography [4], mass spectrometry (MS) [5,6] and nuclear magnetic resonance (NMR) [7,8]). In any case, incorporating a convenient and reliable strategy is of underlying importance for avoiding any metabolic loss, for ensuring reproducibility and for the valid biological interpretation of the data [9,10].

**Figure 1:**
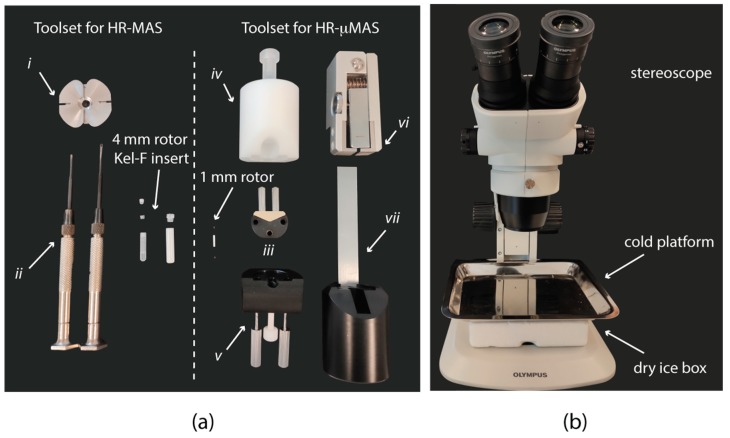
(**a**) Toolsets for (left) high-resolution magic angle spinning (HR-MAS) sample preparation compared to (right) high-resolution micro-magic angle spinning (HR-µMAS) with details of ZrO_2_ rotor of different sizes and packing tools. (i) Clamp tool for opening the rotor cap, (ii) screw drivers for handling Kel-F insert, (iii) µ-rotor holder, (iv) and (v) toolset for closing the µ-rotor caps, (vi) and (vii) toolset for opening the µ-rotor caps. (**b**) Cold workstation and stereomicroscope. A dry ice bucket is placed under a metallic platform, which will be consequently cooled down for allocating the sample manipulations with the µ-rotor under the stereomicroscope.

NMR spectroscopy has been present in the development of metabolomics for decades. Strategies for sample preparations for both the standard high-resolution liquid [7] and high-resolution magic angle spinning (HR-MAS) [11] NMR are well documented and established. The fundamental basis is to minimize the sample exposure to unfavorable conditions (such as contamination, temperature, time, etc.) during the preparation so as to preserve sample integrity and experimental reproducibility. In general, the procedure is straightforward. For example, in HR-MAS with a Bruker 4 mm rotor, it consists of introducing the mg (or µL) level of a sample into a Kel-F bio-insert (Figure 1a) by either a pipette or a biopsy punch, depending on the sample morphology (liquid or semi-solid); if necessary, this is followed by adding sufficient D_2_O (or buffer) to homogenize the content for high-quality data acquisition; and lastly, the insert and the rotor are sealed with the corresponding use of dedicated toolsets (Figure 1). The entire procedure takes about 5–10 min [11,12,13,14,15].

Recently, a new NMR technology, high-resolution micro-magic angle spinning (HR-µMAS), was introduced [16]. It targets the microgram (µg) level of specimens and has shown promising results towards metabolomics [17,18]. HR-µMAS could open a new and unexplored NMR platform [19], such as the possibility of carrying out longitudinal metabolic investigation on living animals. This contributes to the fact that µg scale analysis permits (1) minimal surgical tissue excision and (2) homogenous sampling from a heterogeneous specimen.

However, the sample preparation for HR-µMAS NMR spectroscopy (i.e., with a 1 mm µ-rotor) is a strenuous task [19] compared to the mg sampling with HR-MAS (i.e., 4 mm rotor). As trivial as it may seem at first glance, handling delicate specimens at the µg level requires high precision skills and tools. One has to consider that displacing such a minuscule sample mass (<500 µg) in a confined volume (<500 nL), in a clean and efficient manner, creates an inevitable difference with HR-MAS. Moreover, unlike HR-MAS, different sample morphologies also require different strategies with different toolsets, constituting additional steps for HR-µMAS. 

Presently, the sample preparation for HR-µMAS has yet to be fully described and documented. The main bottleneck lies in the necessary requirements, namely methodical sample collection, feasible sample filling and quick µ-rotor sealing. Few attempts have been made to simplify the preparation of µMAS for metabolomics [20,21,22]. One example is the use of a 1 mm disposable Kel-F µ-rotor along with the concept of sampling insert (i.e. a 4 mm Kel-F bioinsert) using a glass capillary [20]. The intent is to facilitate the sample filling and eliminate the rotor sealing. However, the overall procedure is not efficient due to the limitations of handling a non-rigid Kel-F rotor. Moreover, the use of an insert lowers the filling factor and, hence, lowers the detection sensitivity by, in this case, nearly one-third. In addition, this approach presents a high risk of damaging the MAS stator due to the spinning of a fragile glass capillary insert. As such, new strategies must be explored and attuned to metabolomics studies.

After over 500 sample preparations in a span of two years with µ-rotor, this report summarizes these experiences and outlines the general preparation strategies for different specimens targeting to HR-µMAS NMR metabolomics. The strategies adopt three principal criteria: (1) rapid and clean sampling, (2) direct, clean and consistent sample filling and (3) quick µ-rotor sealing. Although the guidelines herein are based on a JEOL 1 mm µ-rotor, the strategies could provide the basis for designing new procedures for metabolomics studies including with µ-rotors from other manufacturers. 

## 2. HR-µMAS Sample Preparation

As an initial and major modification from HR-MAS, all manipulations with both the sample and the µ-rotor are carried out under a stereomicroscope (Figure 1b) with a large set of high-precision tools (Figure 1a), each with a specific function. For example, a holder (Figure 1a, iii) facilitates all manipulations of the µ-rotor and is an essential tool in all sample filling procedures described below. The use of a holder also prevents a constant contamination on the rotor surface. Specific toolsets are dedicated for closing (Figure 1a, iv and 1a, v) and opening (Figure 1a, vi and 1a, vii) the µ-rotor caps. Since there are different toolset designs for different µ-rotors (e.g., Bruker, JEOL, etc.), the report will omit the descriptions of operating the tools. Formal training from the vendor is recommended.

Due to the small sample volume, <500 nL, even the tiniest contamination is in line with the sample metabolic content. As a result, the unwanted signals will inevitably disrupt the metabolic spectral profile. Therefore, prior to the sample preparation, special attention to the cleanness of the rotor, the toolsets (i.e., contact with the rotor) and even the working space entirely is essential. Supplementary Materials Figure S1 shows an example of a contamination from a cleaning solvent, ethanol, even after a long drying period. Tips: (1) It is strongly recommended to avoid using solvent other than water for cleaning (i.e., rotor, toolsets, workspace); (2) sonicating the µ-rotor in a warm water bath is recommended to assist in eliminating the tiniest residues.

### 2.1. µg Sampling 

As aforementioned, ensuring the sample integrity is crucial [10], and therefore preconditions must be regulated. For example, the time for the samples to be exposed to an unfavorable temperature must be short. Therefore, the availability of dry ice during all procedures is important. While working with the stereomicroscope, one of the complications, the use of a cold platform (Figure 1b) is highly recommended for carrying out the entire sample preparation for keeping the specimens under a favorable environment. Ideally, the entire procedure from sampling to filling would be better performed inside a walk-in cold room facility.

Prior to µg sampling, specific preconditions must be considered for different specimens. For example, intact cells are generally susceptible to their surroundings, and the permeability of the membrane should be considered. In that sense, the pH should be controlled using a buffer [23], as well as the cell concentration in the suspensions; it should be efficient for spectral sensitivity but avoiding a crowding effect causing cell asphyxiation or rupture [24]. In addition, cells are particularly sensitive to the changes in temperature, so the freeze/thawing cycles have to be avoided as the formation of ice crystals will damage the cell membrane [25].

For animal tissues, the presence of excessive blood content distorts spectral resolution due to the existence of paramagnetic species [11]. Unlike in HR-MAS, even a very small content of blood in a 500 µg sample could render a significant paramagnetic effect on the spectrum. Therefore, it is advised to wash the tissue with D_2_O or saline solution (0.9% NaCl w/v) prior to filling. However, caution must be applied to prevent removal of the metabolic content. We recommend a swift immersion into a deuterated saline solution. 

Depending on the sample morphologies, the µg sampling procedure is performed either by a pipette for biofluids or fluid matrices (i.e., intact specimens suspended in a liquid) or by a microsized biopsy punch for tissues. 

Tips: (1) With a pipette, it is recommended to use a micropipette tip with hydrophilic surface (ideally with glass) to avoid surface tensions with the individual specimens; (2) with a microbiopsy punch, it is recommended to cool down the tip to prevent a complete thawing of the tissue during the collection and transferring.

### 2.2. Sample Filling

The sample filling procedure can be considered the most significant step in the sample preparation. This is because of its extensive manipulation of the sample. Unfortunately, it is not straightforward to fill µg specimens into a tiny rotor (with a 0.5 mm inner diameter). It should comply with the following criteria: (1) a good sample homogeneity inside the µ-rotor to achieve high spectral resolution data (i.e., avoiding the presence of air bubbles). For example, the tiniest air pocket can worsen the spectral resolution; (2) a correct sample displacement inside the µ-rotor for maximum sensitivity detection; (3) a sufficient sample mass to achieve a good sensitivity; (4) a good weight balance of the µ-rotor to avoid spinning deficiency; and (5) a repeatable sampling procedure for data reproducibility. Subtle deviations in all these criteria could affect the individual spectral data and diminish both the data repeatability and reproducibility. What follows are the details of three strategies, each with different toolsets targeting different specimens.

#### 2.2.1. Micropipette or Microsyringe

Target samples: biofluids such as serum, plasma, urine and tissue extract.

Pipette tip/fine-needle characteristics: both must be narrow in diameter (i.e., < 0.5 mm) to be able to traverse through the entire µ-rotor length (e.g., Eppendorf GELoader® tips, Hilgenberg glass needles). 

µ-rotor requirements: one closed end (i.e., one end is open while the other is closed with a µ-rotor cap).

Guidelines:

○ Convey 1–2 µL of fluid inside the µ-rotor by placing the tip (or needle) at the bottom. Tip: the µ-rotor is placed in the holder (Figure 1a, iii) to facilitate the handling.
○ Release the fluid slowly while moving upwards to avoid air bubbles.
○ Centrifuge (~3000 rpm, ~30 s; recommended at 4 °C) the filled μ-rotor to ensure the exclusion of air bubbles.
○ Seal the µ-rotor with a designated µ-rotor cap using the dedicated toolset (e.g., Figure 1a). Caution: Ensure a sufficient space for the sealing; if not, the sealing would be impossible.

Estimated time: 5–10 min.

#### 2.2.2. Centrifugal Microfunnel

Target samples: fluid matrix samples such as cells or whole organisms in biofluids (e.g., blood, nematode and microbe). 

Funnel characteristics: biocompatible material (ideally with glass or parylene coating, etc.). Caution: The surface must be smooth and hydrophilic to prevent sample shearing, and to assist the transference into the µ-rotor.

µ-rotor requirement: one closed end.

**Figure 2:**
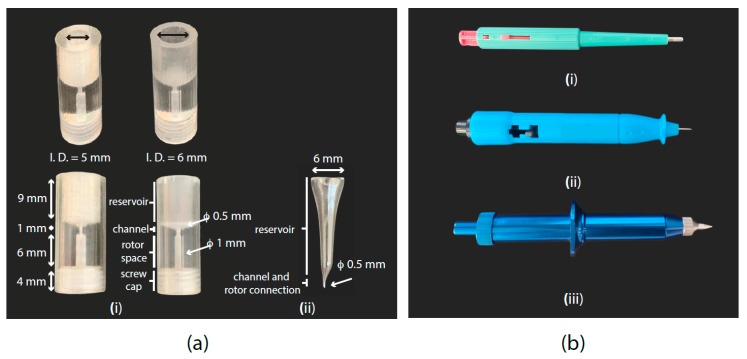
(**a**) (i) 3D-printed funnel. It consists of a bulk polymeric funnel, which leaves a space for the µ-rotor, and a µ-channel connecting the rotor volume with the sample reservoir. After printing, the funnel should be submitted to a coating process with deposited poly(p-xylylene) (i.e., parylene), which adds a layer of 0.5 µm and generates a biocompatible and smoother surface. (ii) Custom-made glass funnel. It connects the funnel reservoir with the µ-rotor through a short channel. Suggested convenient dimensions are shown in the picture for both types of funnel. (**b**) Different biopsy punch models. (i) 2 mm biopsy punch used for sample collection and filling process inside the standard HR-MAS Kel-F insert. (ii) Disposable and (iii) reusable 0.5 mm biopsy punch fitting the inner diameter of the µ-rotor for HR-µMAS to facilitate the filling process.

Guidelines (based on a 3D-printed microfunnel shown in Figure 2a):

○ Place the µ-rotor inside the designated space in the funnel.
○ Convey (by either pipette or glass syringe) the matrix into the funnel reservoir.
○ Centrifuge at 4 °C. The speed and time depend on the funnel materials (polymers such as Kel-F and Teflon allow for faster centrifugation, while glass will only tolerate a gentle centrifugation).
○ Close the µ-rotor.

Estimated time: 10–15 min.

#### 2.2.3. Microbiopsy Punch

Target samples: semi-solids such as animal and food tissues, or cell pellets.

Punch characteristics: sharp edges for clean-cut and reproducibility. The outer diameter must be smaller than or equal to the inner diameter of the µ-rotor (i.e., <0.5 mm, examples in Figure 2b).

µ-rotor requirements: Both ends must be opened to prevent the effect of the air pressure during the filling.

Guidelines:

○ Extract µg sample by punching. Tip: Frozen samples facilitate a clean excision.
○ Fast transfer of the excised sample into the µ-rotor (placed in a holder).
○ Follow by adding a drop of D_2_O (or buffer) into the sample to homogenize and to avoid dehydration. Note: Water content in the sample can have a large effect on the spectral quality (Supplementary Materials Figure S2).
○ Close one end of the µ-rotor followed by a gentle centrifugation (~1500 rpm, ~30 s, at 4 °C) for sample positioning and releasing air bubbles.
○ Fill the remaining µ-rotor volume with D_2_O or buffer. Tip: Use the holder.
○ A second centrifugation should be applied to further homogenize the sample.
○ Close the µ-rotor.

Estimated time: 15–20 min.

### 2.3. Pre-Acquisition Considerations

After the sample filling, a few critical precautions must be applied prior to inserting the µ-rotor into the probe. Under the stereomicroscope, one must carefully inspect the µ-rotor caps to see if they are in good condition (i.e., no sign of damage) to ensure a good and stable sample spinning, and that the caps are tightly fit and secure in the µ-rotor to prevent sample leakage. In addition, the cleanliness on the rotor surface is absolute; any tiny particles (i.e., dried sample residue, dust, etc.) could damage the stator. Hence, for the same reason, it is advised to clean the entire µ-rotor surface, including the caps, with high-quality tissue (e.g., Kimtech wipe) and/or with sticky pens (e.g., standard JEOL RESONANCE Inc. preparation tool set, Supplementary Materials Figure S3) prior to displacing the µ-rotor into the stator. 

**Figure 3:**
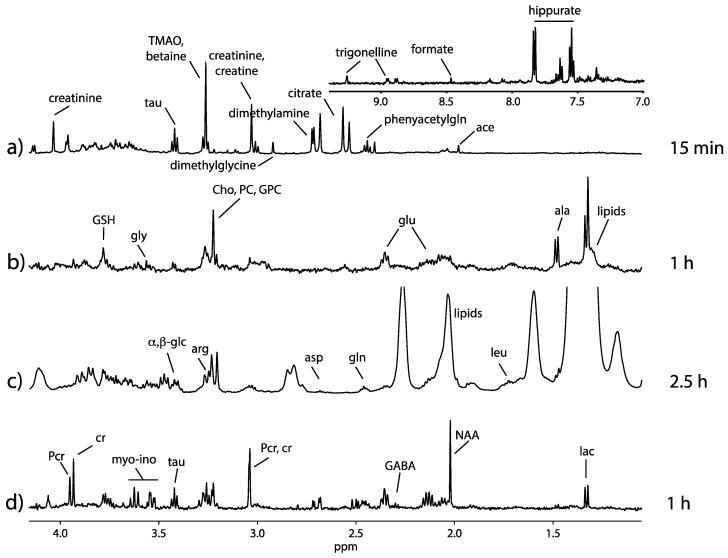
^1^H HR-µMAS nuclear magnetic resonance (NMR) spectra spinning at 4 kHz from (**a**) rat urine in PBS/D_2_O prepared with automatic pipette, (**b**) 400 nL K562 cell suspension in PBS/D_2_O buffer (pH = 7.4) prepared with a 3D-printed funnel, (**c**) 400 nL of *C. elegans* (*n* = 30) suspension in D_2_O prepared with a custom-made glass funnel and (**d**) 500 µg brain tissue prepared with a disposable 0.5 mm biopsy punch. Total acquisition times are indicated for each spectrum. Spectra (**b**) and (**d**) were acquired using the Carr-Purcell-Meiboom-Gill (CPMG) pulse sequence (d20 = 0.2 ms, loop = 200), and spectra (**a**) and (**c**) were acquired using the NOESY pulse sequence (mixing time 0.1 s). The main metabolic signatures are identified on the spectra. The preparation for each specimen is detailed in the Supplementary Protocol S1.

Once the µ-rotor is introduced in the stator, sample spinning must proceed with caution. A manual adjustment to the desired spinning frequency is recommended. Typical spinning rates ranging from 4 to 6 kHz are sufficient to suppress the susceptibility broadening in MAS NMR spectra of semi-solids such as tissues and cells [11]. Such moderate rate prevents the sample temperature from increasing and provides adequate conditions during the data acquisition. As an example, Figure 3 shows excellent spectral quality from a spinning rate of 4 kHz. The spinning side-bands are displaced outside the metabolites’ chemical shift range, and the metabolic isotropic signals are with good resolution.

^2^H-field locking can be difficult due to the low ^2^H content in the sample volume; the signal is often weak and unreliable for field shimming. Consequently, the strategy of field shimming with HR-µMAS is different to that with HR-MAS. Despite the applied MAS shim sets being the same [26], the metabolic signals are generally weak with HR-µMAS to shim with a continuous acquisition; therefore, it is recommended to shim first on the water signal with continuous mode, followed by an incremental acquisition on metabolic signals. Consequently, the shimming can be a long and demanding process; hence, the use of sacrificed sample(s) is strongly recommended. One can also consider adding a small content of a known metabolite (e.g., <1 mM alanine, sucrose) in the sacrificed sample rendering a shimming process with a continuous acquisition. Supplementary Materials Figure S4 shows the resultant signals of the metabolic additive used for shimming. 

Provided the sample filling into a µ-rotor is identical, then the same shim set of the sacrificed sample can be applied, with minor adjustments. However, one should note that a slight deviation in the sample filling, resulting in an air pocket or insufficient water content, can deviate the shims from a sacrificed sample. 

The experimental parameters used in the spectral acquisitions are no different with the standard NMR experiments, except with one exception: the power level for a 90° pulse is low owing to the high B_1_ efficiency with a µ-size coil. 

Figure 3 shows the resultant NMR spectra of different specimens (biofluid, cell matrix, small organism and animal tissue), each prepared by the different strategies described above. The details of the preparations are summarized in the Supplementary Materials (Protocol S1) and should provide the basis for future metabolomic studies with HR-µMAS or with µMAS in general.

## 3. Final Remarks

Acknowledging the difficulties for preparing µg-scale specimens in a specific tiny sampling volume for HR-µMAS NMR spectroscopy, this report presents general guidelines of the sample preparation strategies for the different type of specimens (biofluid, fluid matrix and tissue). Although the basis of these preparations is similar to those for HR-MAS, they are considerably complicated by the fact that the manipulation of the minuscule specimens along with a tiny rotor must be performed in a repeatable, clean and timely manner. A slight deviation could affect the overall data, resulting in non-reproducible data acquisition and consequently in variable or misinterpreted analysis. 

**Figure 4:**
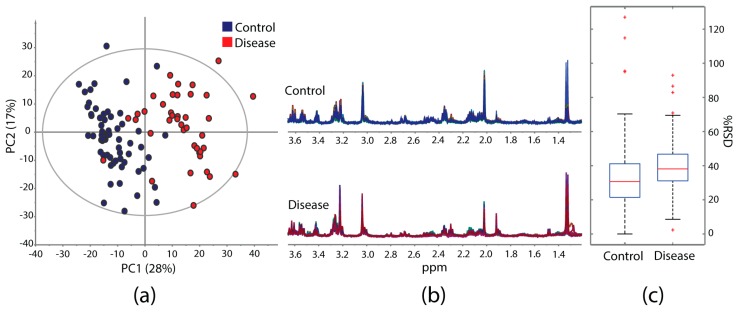
(**a**) PCA scores plot obtained from a model study containing 102 data on brain tissue (control and disease) from an initial 112 samples, where 10 (9%) were discarded due to either extra peaks from contamination or poor spectral quality from air pockets or dryness. Quality parameters: 14 components, R^2^X = 0.85, Q^2^ = 0.63. (**b**) Overlaid ^1^H-HR-μMAS NMR spectra of (blue) 62 control and (red) 40 diseased tissue samples. (**c**) Boxplots of the relative standard deviation (%RSD) values calculated from the individual bucket intensity (*Δ* = 0.04 ppm) of both groups across the spectral region (0.76–5.28 ppm). It summarizes the lower, median and upper quartiles, with the black whiskers displaying the range of data, and the red cross indicating the outlier data points.

The guidelines herein can provide a good basis for designing NMR-based metabolomics studies of µg-scale heterogeneous specimens with HR-µMAS or with µMAS in general. An example is shown in Figure 4; over 100 sampling data on tissue (control and disease) were acquired adopting the guidelines stated in this report. The results offer good data reproducibility for reliable multivariate data analysis. The Principal Component Analysis (PCA) score plots (Figure 4a) clearly display two groups within the data set. Its quality parameters (R^2^X = 0.85, Q^2^ = 0.63) demonstrate the data acquisition is trustworthy where the sample preparation has an important contribution. Figure 4b exhibits good regularity of the NMR spectral profiles within the groups. Acceptable values of the relative standard deviation (%RSD) of the individual spectral bins are found with a median of 30.7% for control and 37.6% for disease (Figure 4c). The slightly higher value of the latter is attributed to the accentuated heterogeneity of the tissue itself.

It should be noted that among the different types of specimens, fluid matrices are the most challenging of all. This is due to the difficulty in achieving a required quantity of intact specimens (e.g., tiny organisms and cells) in a nL scale volume. Although the strategy (centrifugal microfunnel) described above improves the sample filling, it is still a primitive and tedious approach. One should consider redesigning the µ-rotor to incorporate a filtration system that can improve sample filling for fluid matrices, which would be a great advantage. For example, an internal filter inside the rotor would retain the matrix (i.e., specimens), while the fluid would be guided out. This method of collecting and filling the sample would assist in concentrating the specimens inside the µ-rotor and render an increase in the sensitivity of the experiments. In addition, this potential methodology would benefit from the use of microfluidic technology for gently guiding the susceptible specimens inside the µ-rotor.

## Supplementary Materials

The following are available online at https://www.mdpi.com/2218-1989/10/2/54/s1, Protocol S1: Detailed preparation for the samples generating the spectra in Figure 3, Figure S1: Garlic and brain tissue displaying ethanol impurity signals. Figure S2: Differences in the spectral resolution depending on the water content shown in different specimens. Figure S3: µ-rotor handling and cleaning tools. Figure S4: Example of spectrum obtained by adding a known amount of sucrose embedded in a brain tissue sample.
