# Supplementary material for: General Guidelines for Sample Preparation Strategies in HR-µMAS NMR-based Metabolomics of Microscopic Specimens
Source: Metabolites. 2020 Jan 30;10(2):54. doi: 10.3390/metabo10020054 (PMC7073555; doi:10.3390/metabo10020054)
Supplement: Supplementary file 1 [file metabolites-10-00054-s001.pdf]

Supplementary materials

General guidelines for sample preparation strategies in HR-μMAS NMR-based metabolomics of microscopic specimens

Covadonga Lucas-Torres[*,1], Thierry Bernard[1], Gaspard Huber[1], Patrick Berthault[1], Yusuke Nishiyama[2,3], Pancham S. Kandiyal[4], Bénédicte Elena-Herrmann[4], Laurent Molin[5], Florence Solari[5], Anne-Karine Bouzier-Sore[6], Alan Wong[1]

[1] NIMBE, CEA, CNRS, Université Paris-Saclay, CEA Saclay 91191 Gif-sur-Yvette, France; Covadonga.Lperez@gmail.com, thierry.bernard@cea.fr, gaspard.huber@cea.fr, patrick.berthault@cea.fr, alan.wong@cea.fr
[2] JEOL RESONANCE Inc., Musashino, Akishima, Tokyo 196-8558, Japan
[3] RIKEN-JEOL Collaboration Center, Yokohama, Kanagawa 230-0045, Japan; yunishiy@jeol.co.jp
[4] Univ Grenoble Alpes, CNRS, INSERM, IAB, Allée des Alpes, 38000 Grenoble, France; pancham-singh.kandiyal@univ-grenoble-alpes.fr, benedicte.elena@univ-grenoble-alpes.fr
[5] Univ Lyon, Université Claude Bernard Lyon 1, CNRS UMR 5310, INSERM U 1217, Institut NeuroMyoGène, Lyon, France; laurent.molin@univ-lyon1.fr, florence.solari@univ-lyon1.fr.
[6] Centre de Résonance Magnétique des Systèmes Biologiques, CNRS-Université de Bordeaux, UMR5536, Bordeaux, France; anne-karine.bouzier-sore@rmsb.u-bordeaux.fr

Protocol S1. Detailed filling procedures for the spectra shown in Figure 3……………………………..p.1

Figure S1. ^{1}H HR-μMAS spectra of garlic and brain tissue displaying EtOH impurity signals………p.2

Figure S2. ^{1}H HR-μMAS spectra revealing the differences in the spectral quality depending on the water content in different specimens…………………………………………………………………...p.3

Figure S3. μ-rotor handling tools……………………………………………………………………p.4

Figure S4. ^{1}H-HR-μMAS spectrum obtained by adding a known amount of sucrose embedded in a brain tissue sample……………………………..……………………………………………..p.5

Protocol S1. Detailed filling procedures for the spectra in shown in Figure 3:

- Biofluid: rat urine (Figure 3a).

1. Dilute the urine sample with PBS/D_2O buffer (2:1 buffer:urine).
2. Using a 20μL automatic micropipette with a Gel-loading tip, pipette 1-2 μL of urine solution.
3. Release the liquid directly from the bottom of the μ-rotor (one closed end). *Tip*: use the holder (Figure 1a, iii) for handling the μ-rotor.
4. Centrifuge the rotor (~ 30 s, 3000 rpm).
5. Seal the rotor with the second cap.

Estimated time: 10-15 min.

- Fluid matrix: K562 cells suspension (Figure 3b).

1. Suspend the cells in PBS buffer using a glass Pasteur pipette.
2. Allocate the μ-rotor (one closed end) in the dedicated space of a 3D-printed funnel (Figure 2a).
3. Using a glass pipette, transfer the sufficient amount of cells suspension to fill-up the funnel reservoir.
4. Centrifuge gently (~ 30 s, 2000 rpm) to facilitate the deposit of the cells inside the rotor.
5. Carefully detach the funnel from the rotor.
6. Centrifuge a second time to eliminate the air bubbles.
7. Seal the rotor with the second cap.

Estimated time: 15 min.

- Fluid matrix: C. elegans suspension (Figure 3c).

1. Collect the *C. elegans* using a glass Pasteur pipette and suspend them in D_2O.
2. Attach the μ-rotor (one closed end) to a glass custom-made funnel (Figure 2b).
3. Transfer the *C. elegans* suspension with the glass pipette into the funnel reservoir. The procedure is optimized for the transference of approximately 30 individuals.
4. Centrifuge gently (~ 30 s, 1200 rpm).
5. See steps 5-7 from "K562 cells suspension".

Estimated time: 15 min.

- Tissue: rat brain tissue (Figure 3d).

1. Punch on the selected region of the brain tissue using a 0.5-mm disposable biopsy punch (Figure 2b).
2. Release the sample inside the rotor (both sides are opened).
3. Using a μ-syringe, add one drop of PBS/D_2O buffer on top of the rotor and gently tap to help the liquid flow through the rotor/sample.
4. Close one end of the rotor.
5. Centrifuge the rotor (~ 30 s, 2000 rpm).
6. Fill-up the rotor volume using a μ-syringe with PBS/D_2O.
7. Centrifuge a second time.
8. Seal the rotor with the second cap.

Estimated time: 20 minutes.

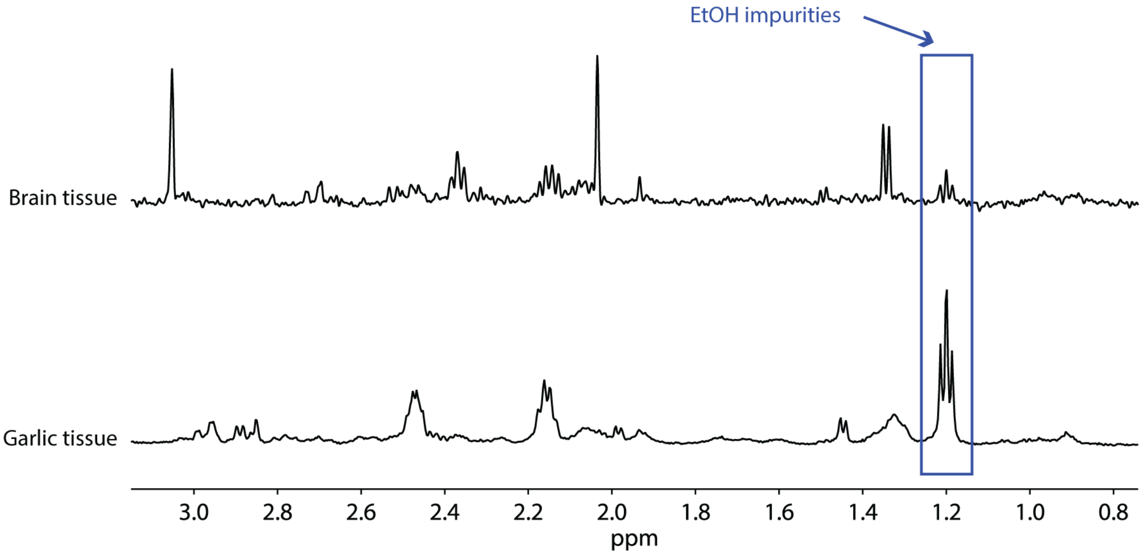

Figure S1. ^{1}H HR-μMAS spectra of garlic and brain tissue displaying EtOH impurity signals. This is the result from cleaning the μ-rotor with ethanol. Even a slightness ethanol content in the μ-rotor would disrupt a spectrum.

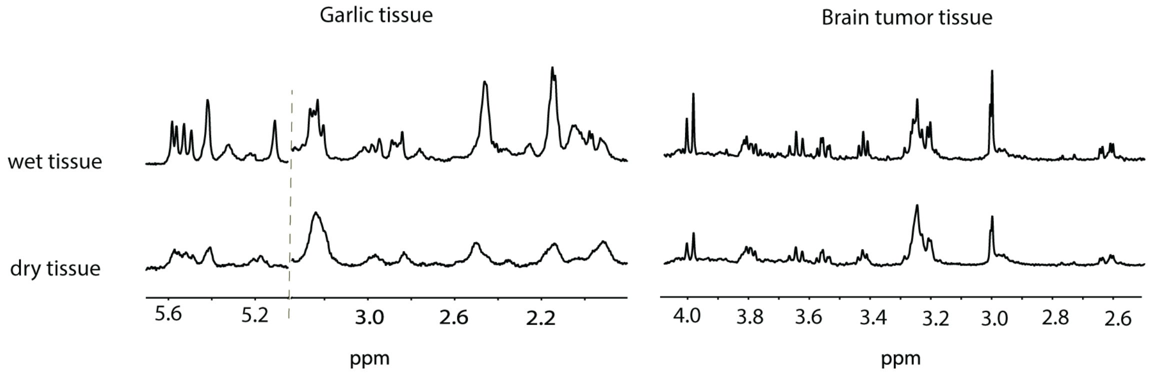

(**a**) (b)

Figure S2. ^{1}H HR-μMAS spectra reveal the differences in the spectral resolution depending on the water content in different specimens: (**a**) Garlic tissue; (**b**) Brain tumor tissue. "Wet" situation refers to the standard procedure were deuterated buffer or D_2O is added to fill up the rotor after the tissue is placed inside. "Dry" situation can be caused by not sufficient solvent been added or sample excessive exposure to air, causing inner dryness.

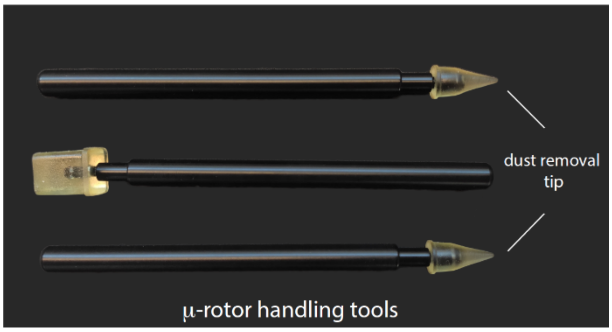

Figure S3. μ-rotor handling tools. The rubber tips help in removing the residual particles on the μ-rotor and caps surfaces (dry residues, dust, etc.).

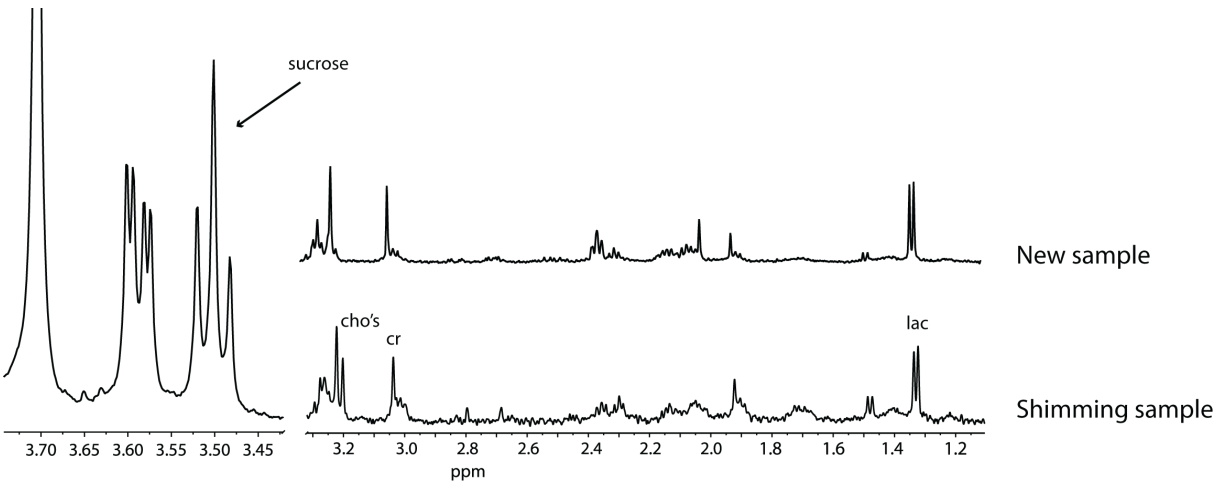

Figure S4. Example of a ^{1}H-HR-μMAS spectrum obtained with sucrose solution added to a brain tissue. This facilitates the field shimming (bottom). The resultant shim sets maintain similar spectral profile and quality on a new sample (top).
